# Improved growth of a child with primary distal renal tubular acidosis after switching from a conventional alkalizing treatment to a new prolonged-release formulation containing potassium citrate and potassium bicarbonate: lessons for the clinical nephrologist

**DOI:** 10.1007/s40620-022-01306-z

**Published:** 2022-03-31

**Authors:** Olivia Boyer, Maria A. Manso-Silván, Sophie Joukoff, Romain Berthaud, Catherine Guittet

**Affiliations:** 1 Néphrologie Pédiatrique, Centre de référence MARHEA, AP-HP, Hôpital Necker-Enfants Malades, INSERM U1163, Institut Imagine, Université Paris Cité, Paris, France; 2 Advicenne SA, Paris, France

**Keywords:** Distal renal tubular acidosis, Plasma bicarbonate, *z*-score, Bone mineral density

## Case presentation

A two-year-old girl presenting with failure to thrive and developmental delay was referred to our department. She had distal renal tubular acidosis (dRTA). Both her weight and height-for-age *z*-scores were < − 3, and skeletal radiography showed thin bones and osteopenia. Calcium-phosphate balance was normal, with a plasma calcium level of 2.4 mmol/L, plasma phosphorus of 1.4 mmol/L, normal parathyroid hormone level (45 ng/L), slightly low 25-hydroxy-vitamin D (19 ng/mL) and normal 1.25-di-hydroxy-vitamin D (79 pg/mL). The insulin-like growth factor I level was also low (42 µg/L).

Treatment was started with sodium bicarbonate 11.9 mEq *b.i.d.*, then increased to 17.8 mEq *b.i.d.* two months later, and continued for approximately two years. Plasma bicarbonate levels were frequently below 17 mmol/L during this period (particularly during the first year) and, although her *z*-scores improved, her height-for-age *z*-score remained < − 3 and her weight-for-age *z*-score remained < − 2. Insulin-like growth factor I was restored to normal levels of 52–60 µg/L with the treatment. Her audiogram progressively declined and she developed sensorineural hearing impairment, needing specialized education. She also presented low bone mineral density (BMD) (spine densitometry *z*-score = − 3.2).

At 4.5 years of age, the girl entered a phase II/III study [1] and was switched to treatment with ADV7103 (Sibnayal)*,* a new product consisting of a combination of potassium citrate and potassium bicarbonate prolonged-release granules and she continued with this treatment throughout a phase III follow-up trial [2]. Dosing was established at 16 mEq *b.i.d.* and was increased to 24 mEq *b.i.d.* four years later to adapt the amount of alkali to her increasing needs as she was growing and gaining weight.

Insulin-like growth factor I level was verified two months after starting treatment with ADV7103 and was normal (117.7 µg/L). Her growth velocity was normal most of the time with this new treatment, including periods above the 97th centile. A summary of plasma bicarbonate and potassium levels, growth and bone mineral density data is provided in Table 1. After two years, she had reached height and weight-for age *z*-scores > − 2. After four years of treatment her bone mineral density was normal (spine densitometry *z*-score = − 1.7). Both growth and bone mineral density parameters remained normal up to the last follow-up visit.

**Table 1:** Summary data for the patient at different periods of her life

Age (years)	Treatment	Plasma bicarbonate	Plasma potassium	Height		Weight		Spine BMD
		mmol/L	mmol/L	cm	*z*-score	kg	*z*-score	*z*-score
Birth	No treatment	NA	NA	42.0	− 3.8	2.3	− 2.3	NA
2.5		12.0–17.0	3.3–3.8	75.0	− 4.5	8.3	− 3.6	NA
3.5	Sodium bicarbonate (for 2 years)	16.7–20.4	3.6–3.9	83.0	− 3.9	9.2	− 3.8	NA
4.5		19.0–21.0	3.7–4.4	91.6	− 3.3	12.4	− 2.5	− 3.2
5.2	ADV7103 for > 5 years)	22.4	3.7	97.5	− 2.6	14.2	− 1.9	NA
6.8		22.6	4.0	111.0	− 1.6	17.2	− 1.6	− 1.9
8.8		22.0	4.2	121.0	− 1.7	23.8	− 0.9	− 1.7
9.8		25.9	3.4	129.3	− 1.3	27.0	− 0.9	− 1.7

*NA* not available

Throughout follow-up with ADV7103, both plasma bicarbonate and potassium levels were normal and nephrocalcinosis remained stable. Clinical examination was normal and her oral skills improved with the use of signs and hearing aids.

## Lessons for the clinical nephrologist

Children with primary dRTA frequently present with stunted growth and failure to thrive (height and weight-for-age *z*-scores < − 2), and low bone mineral density [3, 4]. Bone mineral is pH-sensitive and its solubility increases when bone pH falls below 7. In case of acute acidosis, bicarbonate and phosphate are released from bone as buffers to compensate for metabolic acidosis, with demineralization ensuing through a physicochemical effect [5]. The excess cations, such as calcium, are excreted in urine.

Additionally, chronic metabolic acidosis may induce modifications in bone by stimulating bone resorption and reducing bone formation through a cell-mediated effect. Proton sensing G-protein receptors at the osteoblast level are activated in the presence of high extracellular proton concentrations, which not only has a direct effect on osteoblast function but also regulates complex downstream processes that result in bone resorption by osteoclasts [5].

Our patient had significant metabolic acidosis (plasma bicarbonate = 12.8 mmol/L) and normal plasma anion gap. Urine anion gap was positive, confirming the renal origin of the acidosis. She had nephrocalcinosis, hypercalciuria (urine calcium/creatinine ratio = 1.24 mmol/mmol), and elevated urine pH (6.80), indicating dRTA, later confirmed as hereditary (homozygous *ATP6V1B1* variant) by array comparative genomic hybridization analysis.

Although within the interquartile range (IQR) of age of a large European cohort of patients with primary dRTA [3], the age of the patient at presentation was relatively late, which may have contributed to the severe growth retardation observed. Indeed, in this patient cohort, the median age (IQR) at presentation was 0.5 (0.1–2.5) years, and 0.5 (0.1–1.9) years for the ATP6V1B1 genetic variant in particular [3]. Early diagnosis of dRTA is essential to establish immediate adequate management, and confirmatory genetic testing in patients with a clinical suspicion of primary dRTA is recommended [6]. As progressive sensorineural hearing loss may also occur or develop later in life, the detection and follow-up of hearing problems is also essential to establish educational and intervention programs adapted to the needs of the patient, which is particularly important in hereditary dRTA forms associated with mutations affecting the vacuolar H^+−^ATPase proton-pump, as is the case of our patient [6].

Bone and growth problems arising as a consequence of metabolic acidosis are generally reversible with treatment. Prognosis is generally good in patients with dRTA treated with alkali therapy, but unfortunately, current treatments do not always succeed in restoring normal bone health and growth levels, which may be due to adherence problems or to adequacy of the dose or the dosing scheme [7, 8]. Before treatment, the patient had low serum levels of insulin-like growth factor I, which could have been in part responsible for growth and bone disturbances.

It is important to constantly maintain plasma bicarbonate levels above 20–22 mmol/L (depending on the age of the patient) in order to reach adequate growth and BMD during the patient’s life [3, 6]. This requires selection of an appropriate treatment allowing sustained control of metabolic acidosis, and close monitoring to detect adherence problems or the need for dosing increases in case bicarbonate levels fall.

Through the present case, we report how treatment allowed the patient to get closer to her genetic target stature before the end of puberty. Her estimated adult stature increased from 141.5 cm when she was 4.5 years old to 154.6 cm aged 9.8 years, for a genetic target stature of 165 cm. Mean (± SD) adult height-for-age *z*-score in the European series was − 0.57 ± 1.16 [3] and we still hope that if our patient continues to adhere to her current treatment she will achieve an equivalent adult *z*-score.

Although three doses were initially considered for prescription, only two doses were effectively maintained due to difficulties associated with adherence to treatment caused by bad tolerance and gastrointestinal problems, together with communication and social context difficulties. Due to this unfavorable situation, we could sequentially evaluate the effect on growth and BMD of two different treatments at equivalent alkali doses and twice daily schedules, although the schedule did not follow the recommendations for the standard of care (SoC) [6].

**Fig. 1:**
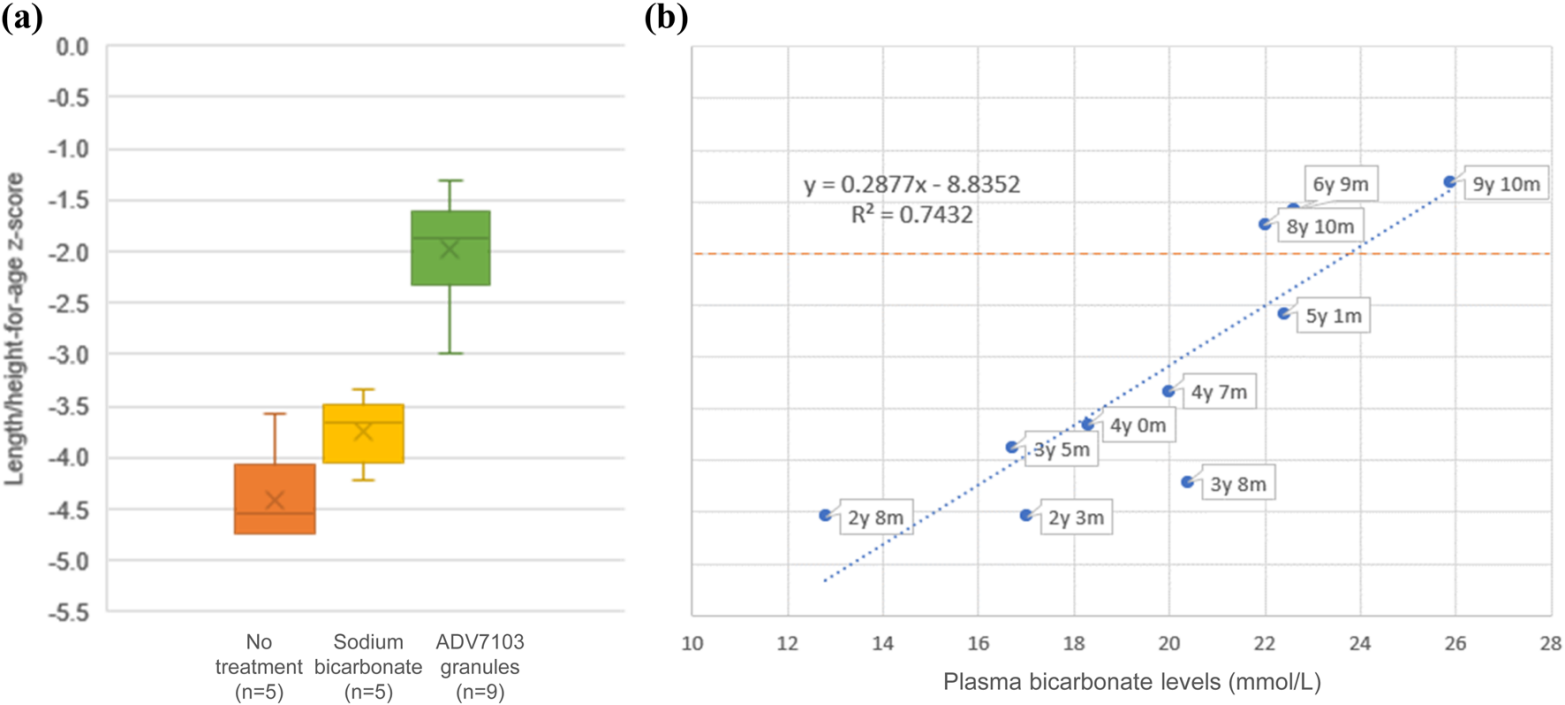
Boxplots representing mean ± SD (squares) and ranges (bars) for length/height-for-age *z*-scores at different periods of the patient’s life (**a**) and correlation between length/height-for-age *z*-scores and plasma bicarbonate levels (**b**)

**Fig. 2:**
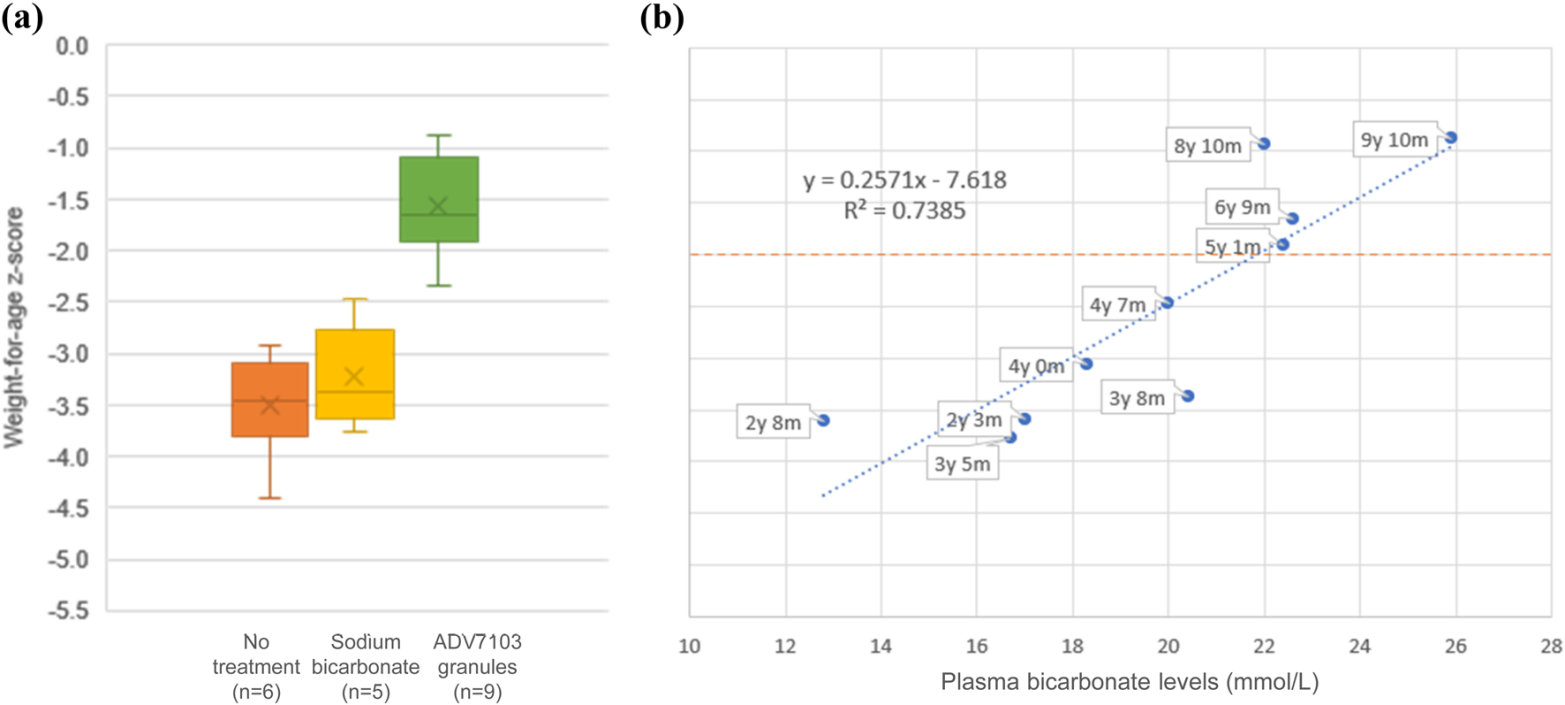
Boxplots representing mean ± SD (squares) and ranges (bars) for weight-for-age *z*-scores at different periods of the patient’s life (**a**) and correlation between weight-for-age *z*-scores and plasma bicarbonate levels (**b**)

As shown in Fig. 1, the mean value of height *z*-scores was 1.8 units higher with ADV7103 (plasma bicarbonate levels ≥ 22 mmol/L) than with SoC (plasma bicarbonate levels rarely > 20 mmol/L), while it was only 0.7 units higher with SoC than without treatment (plasma bicarbonate levels ≤ 17.0 mmol/L). Similar results were obtained for weight-for-age *z*-scores (Fig. 2). As an effect of the alkalizing treatment, urine calcium was reduced to values of 0.20–0.39 mmol/mmol with sodium bicarbonate and of 0.07–0.37 with ADV7103.

The main difference between both treatments is the sustained control during day and night that can be achieved with ADV7103 [9], but not with immediate release sodium bicarbonate twice daily. This can be explained by the pharmacokinetic and pharmacodynamic profiles of both formulations. Oral bicarbonate is rapidly absorbed by the intestinal mucosa and a part of it neutralizes gastric acid in the stomach and is immediately eliminated in the form of CO_2_. Thus, immediate-release sodium bicarbonate twice daily does not achieve sufficient alkali coverage during periods of trough alkali concentration in plasma. Periods of metabolic acidosis can therefore occur, particularly during the night, affecting normal bone homeostasis. This could activate the initial physicochemical response mechanisms of bone to metabolic acidosis, providing bicarbonate and phosphate to the extracellular fluid as direct buffers, and releasing sodium, potassium and calcium, which impacts bone mineral density. It is therefore recommended to give alkalizing treatments as frequently as possible, and daily doses of bicarbonate should be divided into at least 3 or 4 doses [6]. However, this can be difficult in practice and may reduce adherence to treatment.

On the contrary, ADV7103 prolonged-release form is characterized by a continuous dissolution of the alkalizing agents [9] and could maintain reduced differences between peak and trough alkali levels through a flip-flop phenomenon. This could allow sustained control of metabolic acidosis, thus minimizing the periods of activation of bone regulatory mechanisms with twice daily administration. ADV7103 is well-tolerated and reported adherence to treatment is excellent [1], [2].

In conclusion, this case is an example in favor of the importance of early diagnosis of dRTA followed by immediate establishment of adequate treatment. Positive effects on growth and BMD were observed in this patient with ADV7103 when compared to previous treatment, which could be attributed to the improved control of metabolic acidosis with this prolonged-release formulation by virtue of reduced differences between peak and trough alkali levels throughout the day/night.
